# CPPD‐Induced Iliopsoas Bursitis Mimicking Pyomyositis

**DOI:** 10.1002/ccr3.72814

**Published:** 2026-05-25

**Authors:** Hiroki Okunobu, Shinnosuke Fukushima, Hideharu Hagiya, Fumio Otsuka

**Affiliations:** ^1^ Department of General Medicine Okayama University Graduate School of Medicine, Dentistry and Pharmaceutical Sciences Okayama Japan; ^2^ Department of Bacteriology Okayama University Graduate School of Medicine, Dentistry and Pharmaceutical Sciences Okayama Japan; ^3^ Department of Infectious Diseases Okayama University Hospital Okayama Japan

**Keywords:** 16S rRNA, abscess, calcium pyrophosphate deposition disease, culture‐negative, direct sequencing analysis

## Abstract

Calcium pyrophosphate deposition disease may mimic an iliopsoas abscess on imaging. The combined use of polarized light microscopy and 16S rRNA gene analysis can help distinguish crystal‐induced inflammation from infection, thereby preventing unnecessary antimicrobial therapy.

1

A 72‐year‐old woman presented with a 3‐day history of right hip pain and fever. On admission, her temperature was 38.1°C, and physical examination revealed localized warmth and tenderness in the right inguinal region. Laboratory tests revealed a white blood cell count of 13,480/μL and a C‐reactive protein level of 13.93 mg/dL. Contrast‐enhanced computed tomography (CT) revealed a fluid collection in the right iliopsoas muscle (Figure 1A,B). Aspiration yielded purulent fluid, leading to an initial diagnosis of iliopsoas abscess, and treatment with ampicillin/sulbactam was initiated. However, Gram staining, bacterial culture, and specimen‐direct PCR sequencing targeting the 16S ribosomal RNA (rRNA) region failed to identify any pathogen. Polarized light microscopy of the fluid collected from the right iliopsoas muscle similarly revealed calcium pyrophosphate dihydrate crystals (Figure 1C). Based on these findings, she was diagnosed with calcium pyrophosphate deposition (CPPD)‐induced iliopsoas bursitis. Treatment with nonsteroidal anti‐inflammatory drugs led to clinical improvement. Antimicrobial therapy was discontinued after 10 days, and the patient was discharged without recurrence.

**FIGURE 1:**
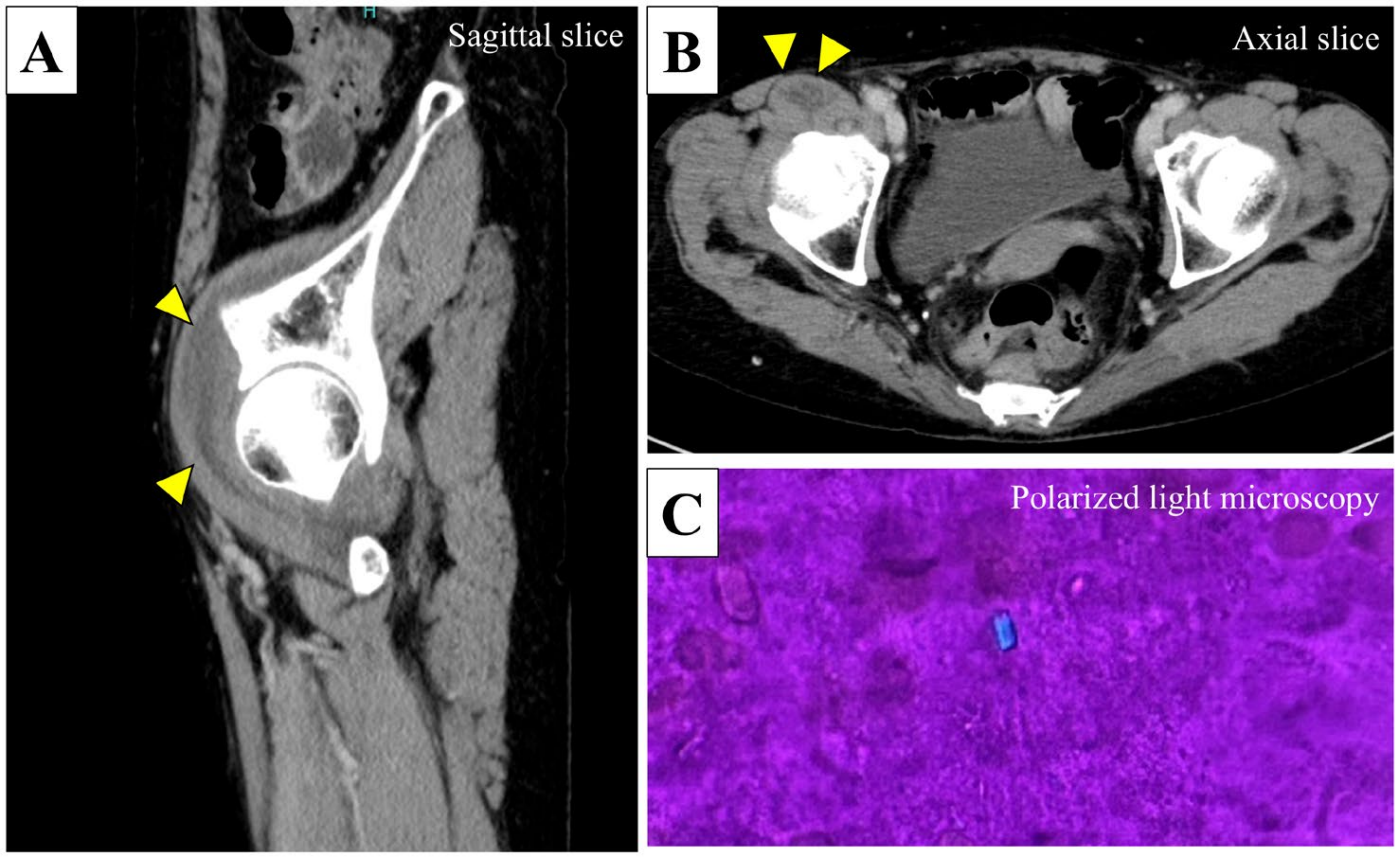
(A, B) Contrast‐enhanced computed tomography shows a well‐defined cystic lesion located anterior to the right hip joint, exhibiting homogeneous low attenuation with peripheral rim enhancement. (C) Polarized light microscopy of the aspirated fluid revealing calcium pyrophosphate dihydrate crystals.

CPPD disease encompasses a spectrum of arthritides that result from the intra‐articular accumulation of calcium pyrophosphate dihydrate crystals. The condition typically presents as acute monoarticular or polyarticular flares of pain and swelling, most commonly affecting the large joints, particularly the knees. In this case, peripheral rim enhancement was observed along the margin of the cystic lesion. Rim enhancement on contrast‐enhanced CT is a characteristic finding of intramuscular lesions such as abscesses; however, it may also be observed as a non‐specific feature in CPPD disease [1]. This diagnostic ambiguity also extends to cases of CPPD disease with superimposed infection. Therefore, differentiation based solely on imaging findings is challenging, and diagnostic aspiration for the detection of CPPD crystals and microorganisms is considered useful. Causative pathogens have been reported to be detected in approximately 80% of abscess specimens [2]; however, since a substantial proportion of cases remain culture‐negative, abscess formation cannot be excluded even when Gram staining and conventional cultures yield negative results. 16S rRNA gene analysis can identify bacterial pathogens undetectable by conventional culture methods [3]. Although infection may coexist with CPPD disease, this case suggests that 16S rRNA can help distinguish CPPD disease from abscess formation. Integrating molecular and microscopic diagnostic approaches can facilitate timely and accurate diagnosis, reducing unnecessary antimicrobial therapy.

## Author Contributions

**Hiroki Okunobu:** writing – original draft. **Shinnosuke Fukushima:** writing – original draft. **Hideharu Hagiya:** writing – review and editing. **Fumio Otsuka:** supervision.

## Funding

The authors have nothing to report.

## Ethics Statement

This case report was reviewed and considered exempt from full Institutional Review Board approval according to our institution's guidelines.

## Consent

Written informed consent was obtained from the patient for the publication.

## Conflicts of Interest

The authors declare no conflicts of interest.

## Data Availability

The datasets used during the current study are available from the corresponding author on reasonable request.
